# Overexpression of *AtBMI1C*, a Polycomb Group Protein Gene, Accelerates Flowering in *Arabidopsis*

**DOI:** 10.1371/journal.pone.0021364

**Published:** 2011-06-20

**Authors:** Wei Li, Zheng Wang, Jian Li, Hongchun Yang, Sujuan Cui, Xiaoxue Wang, Ligeng Ma

**Affiliations:** 1 Hebei Key Laboratory of Molecular Cell Biology, College of Biological Sciences, Hebei Normal University, Shijiazhuang, Hebei, China; 2 National Institute of Biological Sciences, Beijing, China; United States Department of Agriculture, Agricultural Research Service, United States of America

## Abstract

Polycomb group protein (PcG)-mediated gene silencing is emerging as an essential developmental regulatory mechanism in eukaryotic organisms. PcGs inactivate or maintain the silenced state of their target chromatin by forming complexes, including Polycomb Repressive Complex 1 (PRC1) and 2 (PRC2). Three PRC2 complexes have been identified and characterized in *Arabidopsis*; of these, the EMF and VRN complexes suppress flowering by catalyzing the trimethylation of lysine 27 on histone H3 of *FLOWER LOCUS T* (*FT*) and *FLOWER LOCUS C* (*FLC*). However, little is known about the role of PRC1 in regulating the floral transition, although AtRING1A, AtRING1B, AtBMI1A, and AtBMI1B are believed to regulate shoot apical meristem and embryonic development as components of PRC1. Moreover, among the five RING finger PcGs in the *Arabidopsis* genome, four have been characterized. Here, we report that the fifth, AtBMI1C, is a novel, ubiquitously expressed nuclear PcG protein and part of PRC1, which is evolutionarily conserved with Psc and BMI1. Overexpression of *AtBMI1C* caused increased H2A monoubiquitination and flowering defects in *Arabidopsis*. Both the suppression of *FLC* and activation of *FT* were observed in *AtBMI1C*-overexpressing lines, resulting in early flowering. No change in the H3K27me3 level in *FLC* chromatin was detected in an *AtBMI1C*-overexpressing line. Our results suggest that AtBMI1C participates in flowering time control by regulating the expression of *FLC*; moreover, the repression of *FLC* by AtBMI1C is not due to the activity of PRC2. Instead, it is likely the result of PRC1 activity, into which AtBMI1C is integrated.

## Introduction

Polycomb group proteins (PcGs) were first identified in *Drosophila melanogaster*, which maintains the repressed state of homeotic (*Hox*) genes during embryogenesis via histone methylation [1]. The PcGs identified to date form at least two different complexes, Polycomb Repressive Complex 1 (PRC1) and 2 (PRC2), which have repressive functions in flies, humans, and plants [2], [3]. In *Drosophila*, PRC2 is composed of four core proteins, including Enhancer of zeste E(z), an H3K27 methyltransferase, Extra sex comb (Esc), Suppressor of zeste 12 (Su[z]12), and p55. This complex increases trimethyl H3K27 levels, thereby silencing *Hox* gene expression [4] and providing a recruitment site for PRC1 [5]. The PRC1 core in *Drosophila* is composed of Polycomb (Pc), Posterior sex combs (Psc), Ring or Sex comb extra (Sce), and Polyhomeotic (Ph) [6], [7]. The mammalian PRC1 complex contains HPC, HPH, RING1A/B, and BMI1, which are homologs of fly Pc, Ph, Ring (or Sce), and Psc, respectively [2]. Among the RING finger proteins, dRing/Sce and human RING1B have been shown to act as an E3 ubiquitylation ligase that monoubiquitylates lysine 119 in histone H2A [8]. The other two RING domain-containing proteins, RING1A and BMI1, are involved in the positive regulation of RING1B E3 ligase activity; however, they do not possess E3 ligase activity. All three RING domain-containing proteins are required for PcG-mediated silencing [9], [10].

In *Arabidopsis*, there are twelve homologs of *Drosophila* PRC2 subunits, which form three distinct PRC2-like complexes: EMBRYONIC FLOWER (EMF), VERNALIZATION (VRN), and FERTILIZATION INDEPENDENT SEED (FIS). These complexes play important roles in multifaceted developmental processes, including the vegetative phase transition, gametogenesis, embryogenesis and early seed development, and flowering time control [11].

The first PRC2-like complex to be identified and biochemically characterized in *Arabidopsis* was the FIS complex, which prevents endosperm and seed development in the absence of fertilization [12]. The FIS complex, which is composed of MEDEA (MEA), FERTILIZATION INDEPENDENT ENDOSPERM (FIE), FIS2, and MULTICOPY SUPPRESSOR of IRA1 (MSI1) [13], [14], represses the expression of *PHERES1* (*PHE1*) to prevent the central cell from initiating endosperm development before pollination by trimethylating H3K27 in *PHE1* chromatin [15]. Inactivation of the FIS complex by fertilization or the delivery of other unknown factors results in the release of *PHE1* and triggers endosperm development [16].

The EMF complex, which is composed of CURLY LEAF (CLF), FIE, EMF2, and MSI1, functions before and after flowering by targeting different branches of genes for silencing [17], [18]. During vegetative development, the EMF complex suppresses precocious flowering and enables vegetative development by repressing the transcription of *FT* and of the flower MADS box genes *AGAMOUS* and *AGAMOUS-LIKE 19* by mediating the deposition of H3K27me3 at their chromatin [18], [19], [20]. Late during vegetative development and after flowering, CLF in the EMF complex binds directly to the chromatin of the floral repressor *FLC* and its relatives *MAF4* and *MAF5*, leading to H3K27me3 modification and the repression of *FLC* under warm conditions [19]. These results imply that the EMF complex regulates flowering in *Arabidopsis* by repressing the expression of these flowering genes at different stages of development [19], [20].

The VRN complex containing VRN2, CLF, FIE, and MSI1 is another PRC2-like complex that controls flowering and enables *Arabidopsis* to flower after vernalization [11]. Cold exposure or vernalization promotes the formation and targeting of the PHD-VRN complex to *FLC* chromatin. The PHD-VRN complex increases H3K27me3 levels at *FLC* chromatin, leading to sustainable silencing of *FLC* [21]. The repressed state of *FLC*, then, is maintained epigenetically during subsequent plant development until it is reset during embryogenesis [22].

Although several PRC2-like complexes that control *Arabidopsis* development have been reported, PRC1-like complexes were identified only recently, primarily because there is no homolog of *Drosophila Pc* in the *Arabidopsis* genome [5]. The existence of PRC1 in *Arabidopsis* was proposed recently based on evidence showing that the plant chromodomain protein LIKE HETEROCHROMATIN 1 (LHP1) binds H3K27me3 *in vitro* and colocalizes genome-wide with H3K27me3 profiles in euchromatin to turn off gene expression [23].

In *Arabidopsis*, five RING domain-containing PcGs have been predicted [24], and the functions of two RING1 homologs (AtRING1A and AtRING1B) were recently characterized [25]. Severe cotyledon, rosette leaf, shoot apical meristem (SAM), flower morphology, and floral organ identity defects were observed in *Atring1a/Atring1b*, implying that a loss of function of both AtRING1A/B perturbs cell-fate determination [25]. *KNOX* gene release was detected in *Atring1a/Atring1b* leaves, but the level of H3K27 trimethylation at *KNOX* genes was unchanged, indicating that the suppression of *KNOX* genes by AtRING1A/B is independent of the H3K27 trimethylation activity of PRC2 [25]. However, the biochemical function of AtRING1A/B in PcG-mediated *KNOX* gene silencing has not been determined.

There are three BMI1 homologs in *Arabidopsis*: AtBMI1A (At2G30580), AtRING1B (AT1G06770), and AtBMI1C (AT3G23060) [24]. AtBMI1A/B regulate plant embryonic and stem cell development by functioning as E3 ubiquitin ligases and components of PRC1 [26], [27]. Specifically, AtBMI1A/B mediate the ubiquitination of DREB2A in response to water stress, leading to degradation of the protein by the 26S proteasome [26]. In addition, AtBMI1A/B were recently identified as components of PRC1 [27]. AtBMI1A/B mediate H2A monoubiquitination, and some stem cell regulator genes were found to be expressed ectopically in *Atbmi1a/Atbmi1b* cotyledons, implying that AtBMI1A/B are involved in silencing stem cell regulators and sustaining the differentiated state of somatic cells [27]. In comparison, the function of the third BMI1 homolog, AtBMI1C, is unknown.

Here we report that AtBMI1C is a component of a PRC1-like complex and that it exhibits H2A monoubiquitination activity. *AtBMI1C* overexpression causes early flowering in *Arabidopsis* via silencing of the flowering repressor *FLC* and by promoting expression of the flowering activator *FT*.

## Results

### Three AtBMI1s are the homologs of human BMI1, a key component of PRC1

**Figure 1:**
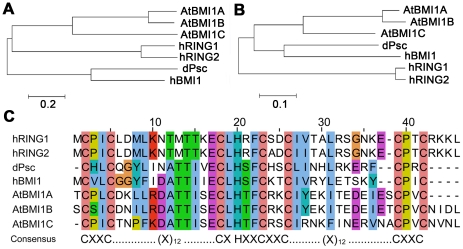
Phylogenetic relationship and conservation of AtBMI1s. (A) Phylogenetic tree based on the full-length sequence of AtBMI1 from *Arabidopsis* and its human and fly homologs. (B) Tree showing the phylogenetic relationships among RING domain-containing AtBMI1s from *Arabidopsis* and their human and fly homologs. (C) Primary sequence alignment of the RING domain from AtBMI1 and its human and fly homologs. The GenBank accessions of the sequences are: NM_128610 (At2g30580) for AtBMI1A, NM_202046 (At1g06770) for AtBMI1B, AY099845 (At3g23060) for AtBMI1C from *Arabidopsis thaliana*, NM_002931 for hRING1, NM_007212 for hRING2 from *Homo sapiens*, and NM_079001 for Psc from *Drosophila melanogaster* (dPsc).

The mammalian PRC1 ubiquitin E3 ligase complex consists of several PcGs, including three RING domain-containing proteins (RING1/RING1A, RING2/RING1B, and BMI1) [8]. To characterize the functions of BMI1 homologs in *Arabidopsis*, we screened a whole-genome *Arabidopsis* sequence database using the protein or RING-domain sequence of Psc from *Drosophila* and its human homolog, BMI1. The existence of three human BMI1-like proteins in *Arabidopsis*, designated AtBMI1A (At2G30580), AtBMI1B (A1G06770), and AtBMI1C (At3G23060), was revealed (Figure 1A); notably, each of these proteins was previously identified as a homolog of BMI1 [24], [27].

The RING domain of the BMI1s is conserved between *Arabidopsis* and animals (Figure 1B and C). The evolutionary conservation of a protein sequence implies functional similarity among distinct organisms. Both the full-length protein and RING-domain sequences of AtBMI1A were closely related to AtBMI1B, but slightly far from AtBMI1C in terms of their evolution (Figure 1A and B), indicating that AtBMI1C may function differently in *Arabidopsis* development. To date, only roles for AtBMI1A/B have been documented; the function of AtBMI1C in plant development remains unknown [27]. Thus, we focused on AtBMI1C in our subsequent experiments.

### AtBMI1C is a ubiquitously expressed nuclear protein

**Figure 2:**
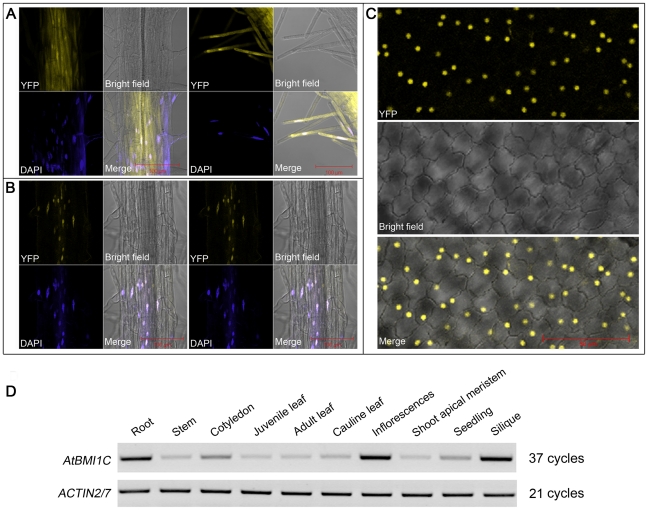
Nuclear localization and expression pattern of *AtBMI1C*. (A) Images of roots from transgenic seedlings harboring YFP driven by the *CaMV35S* promoter. (B) Images of roots from transgenic seedlings harboring YFP-tagged AtBMI1C driven by the *CaMV35S* promoter. Scale bar (red), 100 µm. (C) Images of petals from transgenic plants harboring YFP-tagged AtBMI1C driven by the *CaMV35S* promoter. Scale bar (red), 50 µm. (D) The expression pattern of *AtBMI1C* in seedlings and different organs was analyzed by semiquantitative RT-PCR.

The expression pattern and subcellular localization of AtBMI1C were examined to elucidate the biological functions of the protein. To determine the subcellular localization of AtBMI1C, a reporter gene (Yellow Fluorescence Protein [*YFP*]) was fused to the *AtBMI1C* coding region under the control of the *CAULIFLOWER MOSAIC VIRUS* (*CaMV*) *35S* promoter to generate stable transgenic plants carrying *p35S::AtBMI1C-YFP* or *p35S::YFP* (control). YFP signals were detected in the nucleus and cytoplasm in the roots of *p35S::YFP* transgenic plants (Figure 2A); in comparison, YFP signals in the roots or petals of *p35S::AtBMI1C-YFP* transgenic plants were detected only in the nucleus (Figure 2B and C). Thus, *AtBMI1C* encodes a nuclear-localized protein.

The spatial expression pattern of *AtBMI1C* was examined in various tissues by semiquantitative RT-PCR. Our results indicate weak expression of *AtBMI1C* in the SAM, juvenile leaves, adult leaves, stems, and cauline leaves, and abundant expression in cotyledons, inflorescences, siliques, seedlings, and roots (Figure 2D). Thus, AtBMI1C may function in multiple tissues during development.

### AtBMI1C is a component of PRC1 with H2A monoubiquitination activity

**Figure 3:**
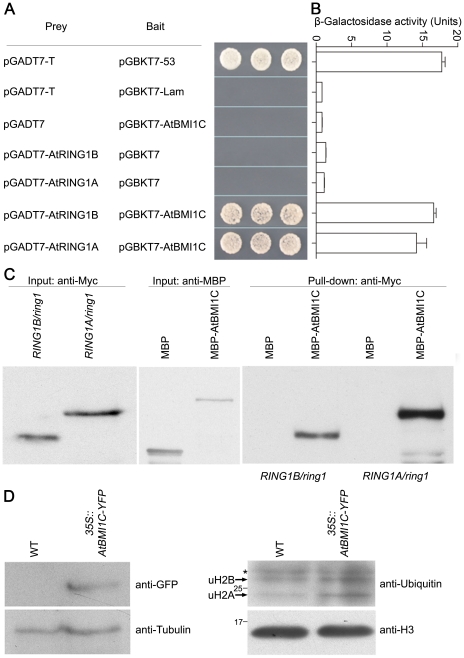
Physical interactions between AtBMI1C and AtRING1A/B, and the detection of H2A monoubiquitination activity. (A) Yeast two-hybrid assay. Positive control: pGADT7-T + pGBKT7-53 (encoding fusions between the GAL4 DNA-BD and AD and murine p53 and SV40 large T-antigen, respectively). Negative control: pGADT7-T + pGBKT7-Lam (encoding a fusion of the DNA-BD with human lamin C; control for interactions between an unrelated protein and either the pGADT7-T control or AD/library plasmid). The indicated combinations of plasmids were co-transformed into the yeast reporter strain, and the interactions of AtBMI1C with AtRINGs were assessed by growth on plates lacking Leu, Trp, His, and adenine. (B) The interactions between AtBMI1C and AtRING1A/B were quantitatively evaluated based on the level of β-galactosidase activity. (C) Pull-down assay. Total protein was extracted from 2 g of eleven-day-old Myc-*RING1A/ring1* or Myc-*RING1B/ring1* plants, respectively. Each protein extract was divided in half and incubated with MBP- or MBP-GST-coated beads. The pulled down fractions were analyzed by Western blotting. (D) Western blot analysis of histone extracts of WT and *35S::BMI3-YFP* using anti-ubiquitin and -H3 antibodies, respectively. Molecular weight (MW) markers (in kDa), monoubiquitinated H2B (uH2B), and monoubiquitinated H2A (uH2A) are indicated. Asterisks indicate cross-reacting bands.

AtBMI1C was localized in the nucleus (Figure 2B and C), similar to AtRING1A and AtRING1B [25]; thus, AtBMI1C was colocalized with AtRING1A and AtRING1B. To address whether AtBMI1C is a component of PRC1, we first explored the interactions of AtBMI1C with AtRING1A and AtRING1B, which are known components of PRC1 in *Arabidopsis* [25], using a yeast two-hybrid assay. AtBMI1C interacted physically with AtRING1A and AtRING1B in yeast (Figure 3B); the N-terminal domain of AtBMI1C, including the conserved RING domain, was required for this interaction (data not shown). The interaction of AtBMI1C with AtRING1A and AtRING1B was further confirmed by a pull-down assay. Both AtRING1A and AtRING1B could be pulled down by AtBMI1C (Figure 3C), suggesting that AtBMI1C and AtRING1 are in the same PRC1 complex.

For a long time, scientists believed that there was no H2A monoubiquitination activity in plants [25]. However, recent data suggest the existence of an AtBMI1A- and AtBMI1B-containing PRC1 complex with H2A monoubiquitination activity [27]. To determine whether AtBMI1C-containing PRC1 possesses H2A monoubiquitination activity, we measured the monoubiquitinated H2A level in wild-type plants and an AtBMI1C-overexpressing line (Figure 3D). Anti-ubiquitin antibodies recognized two specific bands from nuclear histone extracts (Figure 3D); the upper band was recognized by anti-monoubiquitinated H2B (uH2B) antibodies as well (data not shown), suggesting that the upper band was uH2B, while the lower band was monoubiquitinated H2A (uH2A). Moreover, the level of H2A monoubiquitination activity was increased in the AtBMI1C-overexpressing line (Figure 3D). However, there was no obvious change in H2B monoubiquitination activity between the wild-type plants and AtBMI1C-overexpressing lines (Figure 3D). This result suggests that, like its homologs, the AtBMI1C-containing complex exhibits H2A monoubiquitination activity in *Arabidopsis*.

### No loss-of-function T-DNA insertion mutant of *AtBMI1C* was isolated

**Figure 4:**
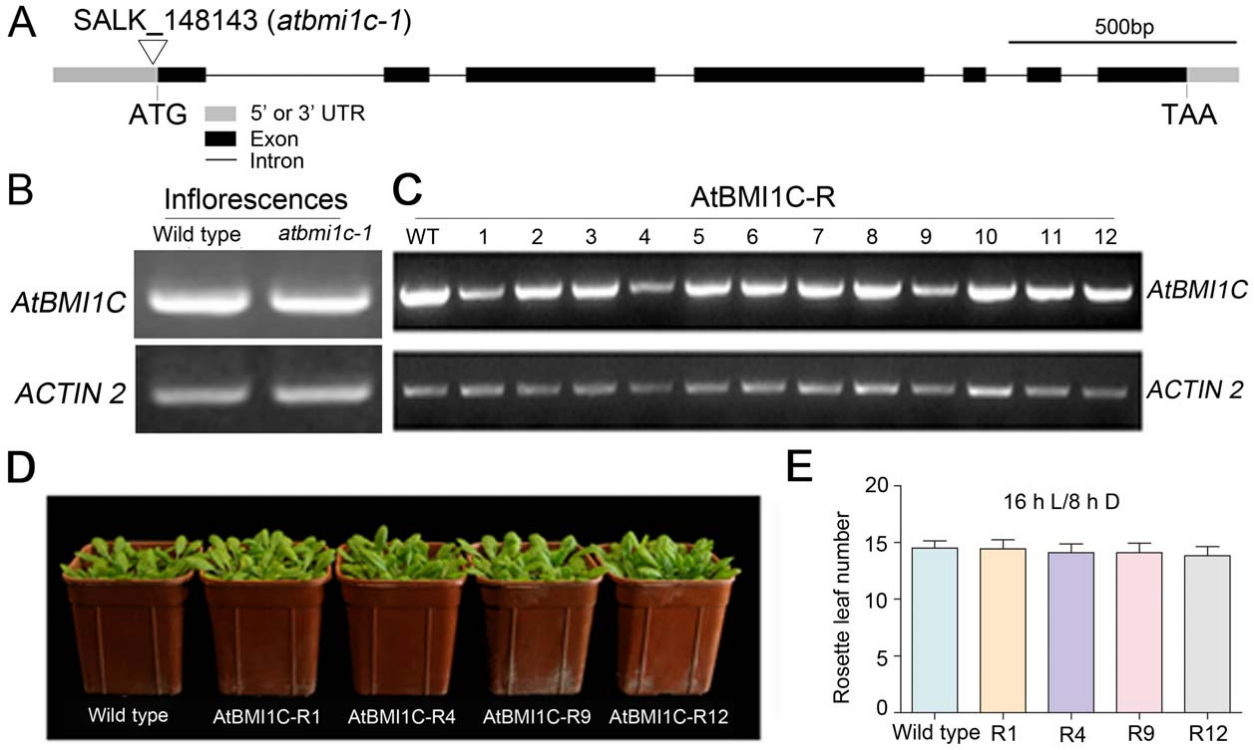
Identification of the *AtBMI1C* mutant and characterization of artificial microRNAi lines. (A) Genomic architecture of *AtBMI1C* and position of the mutation in *atbmi1c-1*. The 5′ or 3′ UTR is represented by a gray bar. Exons are represented by black bars. Introns are represented by black lines. The T-DNA insertion in *atbmi1c-1* (SALK_148143) is located in the 5′ UTR of *AtBMI1C*. Scale bar, 500 bp. (B) Detection of *AtBMI1C* mRNA in a homozygous *atbmi1c-1* T-DNA insertion line by semiquantitative RT-PCR. Total RNA was extracted from the inflorescences of homozygous *atbmi1c* and wild-type plants. Semiquantitative RT-PCR was performed to amplify the full-length transcript using *ACTIN2/7* as an endogenous control. (C) Characterization of *AtBMI1C* mRNA abundance in AtBMI1C-Rs. Total RNA was extracted from the inflorescences of AtBMI1C-Rs and wild-type plants. Semiquantitative RT-PCR was conducted to amplify the full-length transcript using *ACTIN2/7* as an endogenous control. (D) Morphology of the AtBMI1C-Rs, in which *AtBMI1C* was down-regulated, compared to wild type and AtBMI1C-R12, an amiRNAi line in which the expression of *AtBMI1C* was almost the same as in wild type. (E) Flowering time in the AtBMI1C-Rs was the same as in wild type. Plants were grown under LD conditions. The number of rosette leaves was determined after bolting.

To investigate the biological function of AtBMI1C in *Arabidopsis*, T-DNA insertion mutants of *AtBMI1C* were ordered from the *Arabidopsis* Biological Resource Center (ABRC). A homozygous T-DNA insertion allele of *AtBMI1C* was identified (*Atbmi1c-1*, SALK_148143), in which the T-DNA was inserted upstream of the start codon (Figure 4A). However, the T-DNA insertion in *Atbmi1c-1* did not abolish the expression of *AtBMI1C* (Figure 4B). Not surprisingly, no visible phenotype was detected among the homozygous mutant plants (data not shown).

### Lack of obvious defects in down-regulated RNA interference (RNAi) lines of *AtBMI1C*

Because no T-DNA insertion mutant of *AtBMI1C* was identified, RNAi was used to explore the function of AtBMI1C. Three primer pairs were designed using an online program (http://wmd2.weigelworld.org/cgi-bin/mirnatools.pl?page=1), the resulting three constructs were delivered to wild-type plants, and the expression of *AtBMI1C* was analyzed in T1 independent lines. Several transgenic lines (AtBMI1C-Rs) were identified, including AtBMI1C-R1, AtBMI1C-R4, and AtBMI1C-R9, in which the expression of *AtBMI1C* was down-regulated (Figure 4C). Defects, especially in flowering time, were monitored in the next generation; however, no visible phenotype was observed in AtBMI1C-R1, AtBMI1C-R4, and AtBMI1C-R9 compared to control (AtBMI1C-R12) and wild-type plants (Figure 4D and E).

Because no T-DNA insertion mutant was available and because no visible phenotype was observed in our amiRNAi lines of *AtBMI1C*, we next investigated the function of the gene by overexpressing it in *Arabidopsis*.

### *AtBMI1C* overexpression confers an early flowering phenotype in *Arabidopsis*

**Figure 5:**
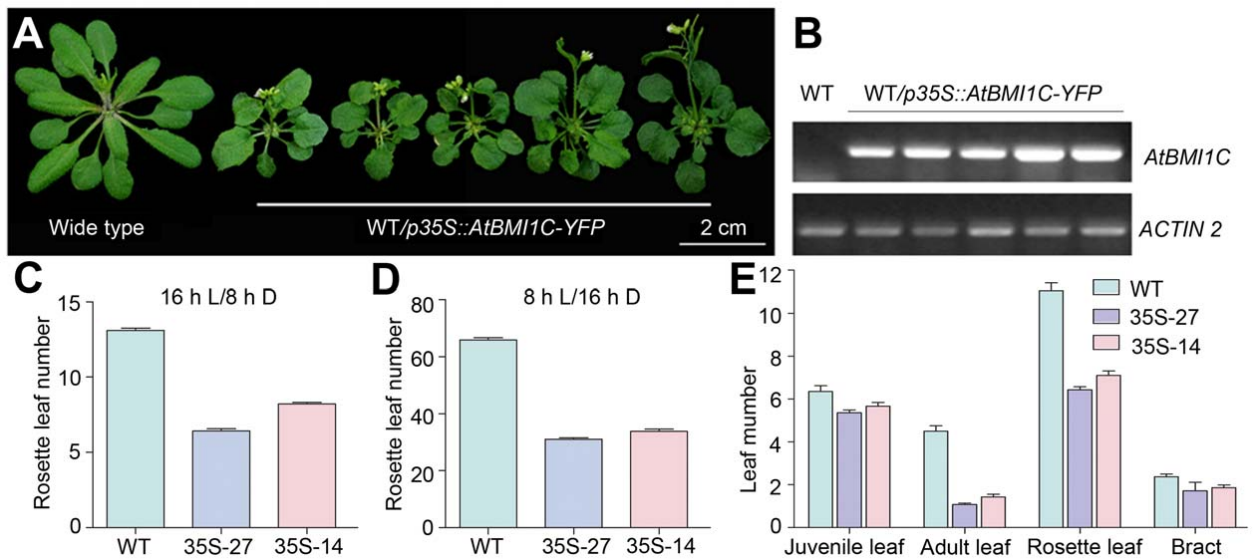
*AtBMI1C* overexpression driven by the *35S* promoter accelerates flowering in *Arabidopsis*. (A) Morphology of *AtBMI1C*-overexpressing plants carrying *p35S::AtBMI1C-YFP* grown under LD conditions for 27 days. The plants flowered earlier than wild type. A total of 30 out of 198 independent T1 lines showed an early flowering phenotype and elevated *AtBMI1C* expression. A number of independent transgenic lines were chosen for the following investigation. Scale bar, 2 cm. (B) *AtBMI1C* expression in transgenic lines carrying *p35S::AtBMI1C-YFP*. *AtBMI1C* expression was measured by semiquantitative RT-PCR. *ACTIN2/7* was used as an internal control. (C) and (D) Determination of flowering time in *AtBMI1C*-overexpressing plants containing *p35S::AtBMI1C-YFP* grown under LD and SD conditions using two *AtBMI1C* overexpressor lines as representatives. The number of rosette leaves was determined after bolting. (E) Vegetative phase transition in *AtBMI1C*-overexpressing plants containing *p35S::AtBMI1C-YFP* grown under LD conditions. Juvenile, adult, rosette, and cauline leaves were counted after flowering. Juvenile and adult leaves were distinguished based on the presence of trichomes on their abaxial surface.

To examine whether the overexpression of *AtBMI1C* affects plant growth and development, we generated *p35S::AtBMI1C-YFP* transgenic plants and monitored their expression of *AtBMI1C* by semiquantitative RT-PCR (Figure 5B). We also recorded the phenotypes of the transgenic lines. The *p35S::AtBMI1C-YFP* transgenic lines showed an early flowering phenotype (Figure 5A). Moreover, using p35S::AtBMI1C-YFP-27 (35S-27) and p35S::AtBMI1C-YFP-14 (35S-14) as representatives, the transgenic lines were found to possess far fewer rosette leaves than wild type under both long-day (LD; 16 h of light/8 h of dark) and short-day (SD; 8 h of light/16 h of dark) conditions (Figure 5C and D and Table 1). In addition, fewer days were required for emergence of the first bud and for opening of the first flower in lines 35S-27 and 35S-14 compared to wild-type plants (Table 1).

**Table 1:** Flowering time in *p35S::AtBMI1C-YFP* plants grown under LD conditions.

Genotype	Days to the first visible bud	Days to the first open flower	Rosette leaf number	n
Wild type	26.00±0.38	32.83±0.38	13.11±0.14	18
*35S*-27	18.43±0.47**	24.57±0.31**	6.43±0.14**	14
*35S*-14	19.61±0.31**	25.78±0.34**	8.22±0.10**	18
*35S*-9	18.62±0.10**	25.62±0.10**	6.58±0.10**	26

Data are presented as the mean ± SD;

**represents a significant difference from wild type (*t*-test, p<0.01);

LD: 16 h of light/8 h of dark.

To exclude the effect of YFP on the function of AtBMI1C, a construct containing only *p35S::AtBMI1C* was transformed into wild-type plants. Flowering time was monitored in the T1 generation and in a number of T2 independent lines. Flowering time in the transgenic plants containing *p35S::AtBMI1C* coincided with that in transgenic plants harboring *p35S::AtBMI1C-YFP*, implying that the early flowering phenotype of the *p35S::AtBMI1C-YFP* transgenic plants was due to the overexpression of *AtBMI1C*, rather than the fusion of YFP to AtBMI1C (data not shown).

*Arabidopsis* undergoes at least two phase transitions during its life cycle: a vegetative and a reproductive phase transition [28]. The vegetative phase transition represents a shift from the juvenile vegetative phase to the adult vegetative phase, which is usually defined by the production of leaves with abaxial trichomes; in comparison, the dramatic vegetative to reproductive phase transition, or floral transition, is characterized by bolting, flowering, and setting seeds for the next generation [28]. To determine the effects of the overexpression of *AtBMI1C* on vegetative and reproductive phase transitions in *Arabidopsis*, we examined the juvenile and adult leaf number in transgenic lines after bolting. There was no significant difference in juvenile leaf number between line 35S-27 or 35S-14 and wild type; however, the adult leaf number in 35S-27 and 35S-14 was dramatically reduced (1.07 and 1.43 adult leaves, respectively) compared with wild type (4.50 adult leaves) (Figure 5E), suggesting that the overexpression of *AtBMI1C* affected only the floral transition and not the vegetative phase transition.

### Tissue-specific *AtBMI1C* expression also produces an early flowering phenotype in *Arabidopsis*

*CaMV35S* is a constitutive promoter with activity in various cell types, tissues, and organs. To investigate the relationship between *AtBMI1C* expression and the phenotype of the transgenic plants, several tissue-specific promoters were selected to drive the expression of *AtBMI1C*, including the *APETALA1* (*AP1*) promoter, a floral primordium-, sepal-, and petal-specific promoter [29].

**Figure 6 pone-0021364-g006:**
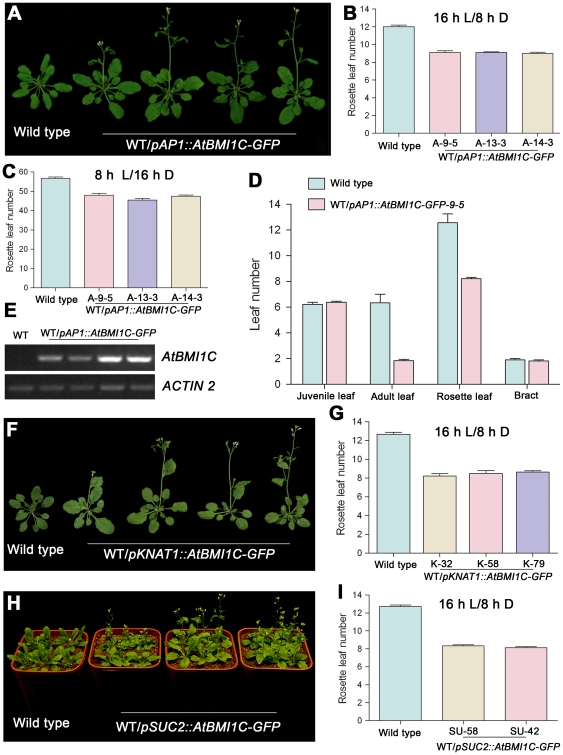
Tissue-specific *AtBMI1C* overexpression promotes flowering in *Arabidopsis*. (A) Morphology of transgenic plants carrying *pAP1::AtBMI1C-GFP* grown under LD conditions for 28 days. The plants showed an early flowering phenotype compared with wild type. A total of 18 out of 72 independent T1 lines showed an early flowering phenotype. A few lines were chosen for the following experiments. (B) and (C) Determination of flowering time in transgenic plants containing *pAP1::AtBMI1C-GFP* grown under LD and SD conditions using three *AtBMI1C* transgenic lines as representatives. The number of rosette leaves was determined after bolting. (D) Vegetative phase transition in transgenic plants containing *pAP1::AtBMI1C-GFP* grown under LD conditions. Juvenile, adult, rosette, and cauline leaves were counted after flowering. Juvenile and adult leaves were distinguished based on the presence of trichomes on their abaxial surface. (E) *AtBMI1C* expression in transgenic lines carrying *pAP1::AtBMI1C-YFP*. Total RNA was extracted from leaves of *pAP1::AtBMI1C-GFP* and wild-type plants. *AtBMI1C* expression was measured by semiquantitative RT-PCR using *ACTIN2/7* as an internal control. (F) Morphology of transgenic plants carrying *pKNAT1::AtBMI1C-GFP* grown under LD conditions for 28 days. The plants showed an early flowering phenotype compared with wild type. A total of 20 out of 108 independent T1 lines showed an early flowering phenotype. (G) Determination of flowering time in transgenic plants containing *pKNAT1::AtBMI1C-GFP* grown under LD conditions. The number of rosette leaves was determined after bolting. (H) Morphology of transgenic plants carrying *pSUC2::AtBMI1C-GFP* grown under LD conditions for 28 days. The plants showed an early flowering phenotype compared with wild type. A total of 24 out of 108 independent T1 lines showed an early flowering phenotype. (I) Determination of flowering time in transgenic plants containing *pSUC2::AtBMI1C-GFP* grown under LD conditions. The number of rosette leaves was determined after bolting.

*pAP1::AtBMI1C-GFP* was generated and introduced to wild-type plants. A total of 18 out of 72 independent transgenic lines harboring *pAP1::AtBMI1C-GFP* exhibited an early flowering phenotype under LD and SD conditions (Figure 6A-C and Table 2). In the mean time, the level of *AtBMI1C* expression in the *pAP1::AtBMI1C-GFP* transgenic plants was measured by semiquantitative RT-PCR. Elevated *AtBMI1C* expression was detected in the early flowering transgenic plants (Figure 6E), indicating that the alteration in flowering time was caused by the overexpression of *AtBMI1C*.

**Table 2:** Flowering time in *pAP1::AtBMI1C-GFP* plants grown under LD conditions.

Genotype	Days to the first visible bud	Days to the first open flower	Rosette leaf number	n
Wild type	28.48±0.23	34.67±0.32	12.00±0.19	27
*AP1*-9	23.00±0.39**	31.00±0.35**	9.11±0.20**	19
*AP1*-13	22.87±0.16**	31.00±0.18**	9.10±0.09**	20
*AP1*-14	22.54±0.21**	30.00±0.30**	9.00±0.13**	24

Data are presented as the mean ± SD;

**represents a significant difference from wild type (*t*-test, p<0.01);

LD: 16 h of light/8 h of dark.

We further investigated the impact of the overexpression of *AtBMI1C* using the *AP1* promoter on vegetative and reproductive phase transitions in a *pAP1::AtBMI1C-GFP*-9 transgenic line (A-9). Our results were similar to those obtained for lines 35S-27 and 35S-14. The juvenile leaf number in A-9 was almost the same as in wild type; however, the adult leaf number in A-9 (1.83 adult leaves) was lower than that in wild type (6.33 adult leaves) (Figure 6D). Thus, the *AP1*-driven overexpression of *AtBMI1C* affected only the floral transition, and not the vegetative phase transition.

### *FLC* suppression and *FT* activation in the *AtBMI1C*-overexpressing lines

*FLC* is a central floral repressor that blocks the expression of floral activators such as *FT* and *SUPPRESSOR OF OVEREXPRESSION OF CO1* (*SOC1*) to prevent the initiation of flowering during vegetative development. The down-regulation of *FLC* activates *FT* and *SOC1* and promotes flowering [30].

**Figure 7:**
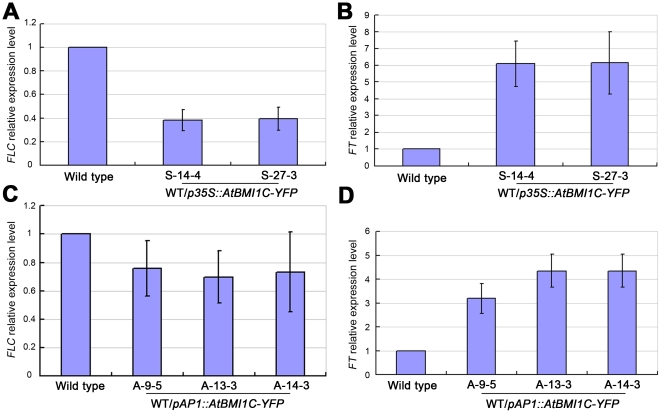
Expression of *FLC* and *FT* in *AtBMI1C*-overexpressing lines. (A) Expression of *FLC* in *AtBMI1C*-overexpressing lines harboring *p35S::ATBMI1C-YFP* as determined by quantitative real-time RT-PCR. (B) *FT* expression in *AtBMI1C*-overexpressing lines harboring *p35S::AtBMI1C-YFP* as determined by quantitative real-time RT-PCR. (C) *FLC* expression in transgenic lines harboring *pAP1::AtBMI1C-GFP* as determined by quantitative real-time RT-PCR. (D) *FT* expression in transgenic lines harboring *pAP1::AtBMI1C-GFP* as determined by quantitative RT-PCR. Total RNA was isolated from ten-day-old transgenic or wild-type seedlings. *FLC* or *FT* expression was measured by quantitative real-time RT-PCR using *ACTIN2/7* as an endogenous control. The values are the mean ± SD of three independent experiments.

To explore the molecular mechanisms responsible for the change in flowering time in our *AtBMI1C*-overexpressing lines, *FLC* and *FT* expression was examined by quantitative RT-PCR. The expression of *FLC* in lines 35S-14 and 35S-27 was 2.5 times lower than that in wild type (Figure 7A). As a result of the down-regulation of *FLC*, the expression of *FT* in lines 35S-14 and 35S-27 was about five times higher than that in wild type (Figure 7B). Similarly, the repression of *FLC* in lines A-9, A-13, and A-14 was also observed (Figure 7C). An increase in *FT* expression of 3-4.5 times in lines A-9, A-13, and A-14 was detected (Figure 7D). These results suggest that *AtBMI1C* overexpression promotes flowering by repressing *FLC* expression and activating *FT* expression.

### Early flowering caused by *AtBMI1C* overexpression in the SAM and vascular companion cells

*FLC* is expressed mainly in the SAM and vascular companion cells [31]. Our initial results indicated that *AtBMI1C* overexpression repressed *FLC* and raised the expression of *FT* to promote flowering. Thus, we hypothesized that expressing *AtBMI1C* in specific tissues, including the SAM and vascular companion cells, would cause early flowering. To test this hypothesis, *KNAT1*, an SAM-specific promoter [32], and *SUC2*, a vascular companion cell-specific promoter [33], were selected to drive the tissue-specific expression of *AtBMI1C*.

Flowering time in T1 and T2 lines expressing *pKNAT1::AtBMI1C-GFP* and *pSUC2::AtBMI1C-GFP* was examined. As expected, *AtBMI1C* overexpression in either the SAM or vascular companion cells caused early flowering. Lines K-32, K-58, and K-79 containing *pKNAT1::AtBMI1C-GFP* flowered earlier (8.22, 8.50, and 8.63 rosette leaves) than wild type (12.65 rosette leaves) (Figure 6F and G and Table 3). Similarly, lines SU-58 and SU-142 carrying *pSUC2::AtBMI1C-GFP* produced fewer rosette leaves (8.33 and 8.12, respectively) compared to wild type (12.71 leaves) (Figure 6H and I and Table 4).

**Table 3:** Flowering time in *KNAT1::ATBMI1C-GFP* plants grown under LD conditions.

Genotype	Days to the first visible bud	Days to the first open flower	Rosette leaf number	n
Wild type	27.00±0.35	33.83±0.29	12.65±0.22	18
*K-*32	22.28±0.34**	28.78±0.34**	8.22±0.25**	18
*K-*58	23.50±0.60**	30.08±0.58**	8.50±0.30**	12
*K-*79	20.47±0.29**	27.11±0.25**	8.63±0.14**	19

Data are presented as the mean ± SD;

**represents a significant difference from wild type (*t*-test, p<0.01);

LD: 16 h of light/8 h of dark.

**Table 4:** Flowering time in *pSUC2::AtBMI1C-GFP* plants grown under LD conditions.

Genotype	Days to the first visible bud	Days to the first open flower	Rosette leaf number	n
Wild type	25.43±0.33	32.29±0.35	12.71±0.16	14
*SU*-58	20.22±0.22**	26.78±0.26**	8.33±0.11**	18
*SU*-142	20.35±0.24**	27.13±0.27**	8.12±0.12**	17

Data are presented as the mean ± SD;

**represents a significant difference from wild type (*t*-test, p<0.01);

LD: 16 h of light/8 h of dark.

### H3K27me3 and H3K4me3 modifications at *FLC* chromatin were unchanged in our *AtBMI1C* overexpressor line

**Figure 8:**
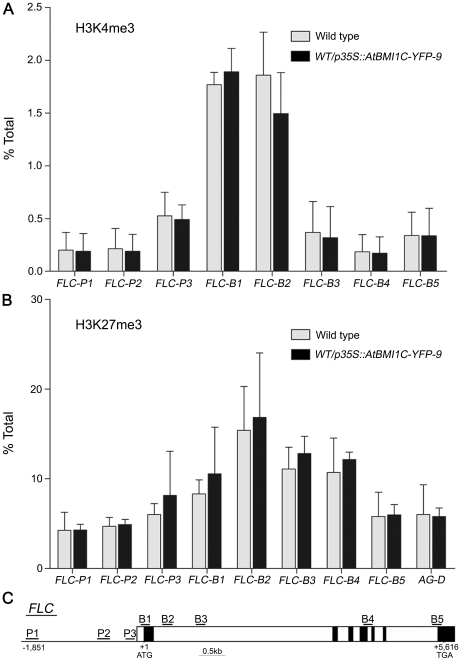
ChIP analysis of H3K4me3 and H3K27me3 at *FLC* chromatin. (A) H3K4 trimethylation levels at *FLC* chromatin between wild type and an *ATBMI1C*-overexpressing line harboring *p35S::AtBMI1C-YFP*. (B) H3K27 trimethylation levels at *FLC* chromatin between wild type and *AtBMI1C*-overexpressing lines harboring *p35S::AtBMI1C-YFP*. Anti-H3K4me3 or -H3K27me3 antibodies were used for the assay. The locations of the primer pairs used to amplify *FLC* fragments across the region are indicated in (C). The values are the mean ± SD of three independent experiments.

Histone modification at *FLC* chromatin plays an essential role in regulating *FLC* [34], [35], [36]. The H3K27me3 or H3K4me3 modification of *FLC* chromatin is related to its repression or activation, respectively [34]. To investigate the biological mechanisms underlying the down-regulation of *FLC* in our *AtBMI1C*-overexpressing lines, we performed chromatin immunoprecipitation analysis to determine the level of H3K27 and H3K4 trimethylation at *FLC* chromatin in *AtBMI1C*-overexpressing line 35S-9. There was no obvious difference in the level of H3K27me3 or H3K4me3 across *FLC* chromatin between wild type and line 35S-9 (Figure 8).

## Discussion

The PcG proteins in PRC2 complexes (e.g., the EMF complex and VRN complex), which are involved in the switch from vegetative to reproductive development, have been documented, and they have been shown to be involved in the H3K27me3 modification of *FT* and *FLC* chromatin [11]. However, little is known about the impact of PRC1 components on the regulation of flowering, even though PRC1 components AtRING1A/B and AtBMI1A/B play essential roles in the repression of plant developmental regulators in *Arabidopsis* [25], [27].

### AtBMI1C is involved in flowering time control in *Arabidopsis*

Genetic and molecular analyses have uncovered multiple signaling pathways that integrate environmental and developmental cues to control flowering time in *Arabidopsis*, including the autonomous, photoperiod, vernalization, gibberellin, and PAF1 complex pathways [37]. PRC2 complexes are involved in cold-induced flowering regulation [34], [37]; however, no PRC1 component has been shown to act as a floral transition regulator.

Because no loss-of-function *AtBMI1C* mutant was available until now, and given that no visible phenotype was observed in our RNAi lines (Figure 4), we generated several transgenic lines in which *AtBMI1C* was overexpressed (*35S-AtBMI1C-YFP*, *AP1-AtBMI1C-GFP*, *KNAT1-AtBMI1C-GFP*, and *SUC1-AtBMI1C-GFP*) in order to dissect the functions of AtBMI1C in *Arabidopsis*. Flowering was accelerated in all *AtBMI1C* overexpressors (Figures 5 and 6). *FLC* is a central repressor of flowering that integrates endogenous signals from the autonomous and vernalization pathways with environmental cues [37]. The repression of *FLC* and activation of *FT* were detected in our *AtBMI1C* overexpression lines (Figure 7). These results suggest that AtBMI1C is involved in flowering regulation. Further analysis using an *AtBMI1C* knock-out mutant will provide additional evidence for the functions of *AtBMI1C*.

### AtBMI1C is a PRC1 component, and the AtBMI1C-containing complex exhibits H2A monoubiquitination activity

AtBMI1C is a homolog of the RING-domain proteins AtBMI1A/B in *Arabidopsis*, Psc in *Drosophila*, and BMI1 in humans (Figure 1B and C). In this report, we found that AtBMI1C interacts physically with AtRING1A/B (Figure 3B and C), indicating that AtBMI1C is a novel component of PRC1 in *Arabidopsis*.

In animals, the repression of gene expression is maintained by PRC1, which monoubiquitinates H2A [8]–[10]. However, H2A monoubiquitination was not detected in plants until a recent report showing that AtBMI1A/B are required for H2A monoubiquitination activity [27]. AtBMI1A/B-mediated H2A monoubiquitination activity has been shown *in vivo* and *in vitro*, and defects in AtBMI1A/B have been shown to impair the production of uH2A [27]. In the present study, we found that an increase in the level of AtBMI1C raised H2A monoubiquitination activity (Figure 3D). Thus, our result supports the notion that AtBMI1-containing PRC1 has H2A monoubiquitination activity in *Arabidopsis*.

### AtBMI1C-containing PRC1 suppresses *FLC* expression independent of H3K27 methylation

PRC1 controls gene expression by altering the level of H2A monoubiquitination [8]–[10], while PRC2 methylates histone H3 at lysine 27 to silence gene expression [2]–[4]. In the present study, we found that AtBMI1C overexpression accelerated flowering and repressed the expression of *FLC* (Figures 5–7). In addition, the repression of *FLC* by AtBMI1C is independent of the level of H3K27 and H3K4 methylation (Figure 8). Therefore, AtBMI1C may function as part of the PRC1 complex to suppress the expression of *FLC* and promote flowering in *Arabidopsis*. However, we cannot rule out the possibility that *FLC* is not a direct target of AtBMI1C-containing PRC1 at this stage. Further study is needed to resolve this issue.

In conclusion, a novel PcG in PRC1, AtBMI1C, was characterized in this study. AtBMI1C is a universally expressed nuclear protein that participates in flowering time control by regulating the expression of *FLC*. The down-regulation of *FLC* was not due to PRC2 activity; rather, it was likely the result of PRC1 activity, which is associated with AtBMI1C.

## Materials and Methods

### Plant materials and growth conditions

*Arabidopsis thaliana* plants (ecotype Columbia [Col–0]) were used in this study. Seeds were surface-sterilized with 2.25% NaHClO and plated on 1X Murashige and Skoog (MS) basal salt medium containing 0.3% agar and 1% (weight/volume) sucrose. After stratification in the dark at 4°C for two days, the plates were transferred to a growth chamber (Percival Scientific) set to 150 µmol m^−2^ s^−1^. The plants used for the flowering time determination were grown in a greenhouse at 22°C (100 µmol m^−2^ s^−1^ cool white fluorescent light) or 18°C (dark). The seedlings sampled for the assay were grown under different conditions as indicated.

### Flowering time determination

For flowering time measurement, mutant and wild-type (Col–0) plants were grown under long-day (LD; 16 h of light/8 h of darkness at 22°C) or short-day (SD; 8 h of light/16 h of darkness at 18°C) conditions.

### Isolation of the *AtBMI1C* T-DNA insertion mutant

T-DNA insertion mutants of *ATBMI1C* (At3G23060) were ordered from the *Arabidopsis* Biological Resource Center (ABRC). Primers specific for sequences upstream and downstream of the T-DNA insertion were designed that could amplify the gene fragment without the T-DNA insertion. To amplify the T-DNA insertion, the T-DNA-specific primer LBb1 (5′-GCGTGGACCGCTTGCTGCAACT-3′) and a gene-specific primer were used. We used two combinations of primers, each consisting of gene-specific primers, and a combination of a gene-specific primer and T-DNA-specific primer to identify individuals that were homozygous or heterozygous for the T-DNA insertion. The position of the T-DNA insertion was determined by sequencing those products carrying T-DNA-genome junctions.

### amiRNA interference (amiRNAi)

Three primer pairs were designed through the web (http://wmd2.weigelworld.org/cgi-bin/mirnatools.pl?page=1) for use in creating amiRNAi constructs according to a previously published protocol [38]. The resulting three constructs were delivered to wild-type plants via *Agrobacterium*-mediated transformation to generate *AtBMI1C* amiRNAi lines (AtBMI1C-Rs).

### Vector construction and transformation

The coding sequence of *AtBMI1C* was amplified from the cDNA of wild-type (Col-0) plants using the primer pair *AtBMI1C-SpeI-F/AtBMI1C-BamHI-R*. The resulting product, *AtBMI1C*, was cloned into pEASY-Blunt using *Spe*I and *Bam*HI and sequenced. Next, the fragment was cloned into the binary vector *pCambia1300* harboring the *CAULIFLOWER MOSAIC VIRUS* (*CaMV*) *35S* constitutive promoter and Yellow Fluorescent Protein (*YFP*) gene using *Xba*I and *Bam*HI to produce the construct *p35S::AtBMI1C-YFP*. *P35S::AtBMI1C* without the YFP tag was also constructed to assess the effects of YFP on the function of AtBMI1C. Constructs for the expression of GFP-tagged AtBMI1C driven by the *AP1*, *SUC2*, and *KNAT1* promoters were also generated (*pAP1::AtBMI1C-GFP*, *pSUC2::AtBMI1C-GFP*, and *pKNAT1::AtBMI1C-GFP*, respectively). The lengths of the promoters were chosen according to the indicated reference.

To create stable transgenic materials, wild-type (Col-0) plants were transformed using *Agrobacterium tumefaciens* GV3101 [39]. Independent transgenic lines were obtained on selective MS medium containing hygromycin. T2 or T3 lines were used for flowering time determination and other assays.

### Semiquantitative RT-PCR and quantitative real-time RT-PCR

About 100 mg of ten-day-old seedlings grown under LD conditions were ground in liquid nitrogen. Total RNA was extracted from the seedlings using Takara RNAiso Plus (D9108A) according to the manufacturer's protocol. The RNA was treated with RQ1 RNase-Free DNase (Promega, M6101) to remove any contaminating DNA.

Three micrograms of total RNA were used for the synthesis of full-length first strand cDNA with a RevertAid First Strand cDNA Synthesis Kit (Fermentas, K1622) according to the manufacturer's protocol. One microliter of cDNA was utilized for semiquantitative RT-PCR.

Quantitative real-time RT-PCR was performed using Takara SYBR Premix Ex Taq in a 7500 fast real-time PCR instrument (Applied Biosystems). The assays were done according to the manufacturer's instructions. *ACTIN2/7* was used as an endogenous control.

### Observation of YFP fluorescence by confocal microscopy

*P35S::ATBMI1C-YFP* transgenic lines (T3) were generated for the subcellular analysis of *AtBMI1C*. A Zeiss Meta confocal microscope was used to detect YFP fluorescence in the roots of ten-day-old seedlings and petals. Images in the YFP, DAPI, and brightfield channels were acquired. The final images were visualized using LSM 510 software.

### Yeast two-hybrid analysis

cDNA from *AtBMI1C* and *AtRING1A* or *B* were cloned into *pGADT7* or *pGBKT7* and co-transformed into yeast strain AH109. Transformation, yeast growth, and quantitative β-galactosidase assays were conducted according to the protocols in the Clontech Yeast Protocols Handbook.

### Protein pull-down assay

AtBMI1C was amplified and cloned into the bacterial expression vector pMAL-c2X (NEB) using *Bam*HI and *Sal*I. The constructs were transformed into BL21 plus competent cells for protein expression. The proteins were induced overnight at 16°C. MBP and MBP-AtBMI1C were purified using amylase resin (NEB). Plant total protein was extracted using extraction buffer (50 mM Tris-HCl [pH 7.6], 150 mM NaCl, 2 mM EDTA [pH 8.0], 1 mM PMSF, and complete protease inhibitor cocktail) then mixed with protein-coated beads and incubated for 2 h at 4°C. The beads were then washed three times with extraction buffer. The bound proteins were eluted with SDS-PAGE sample loading buffer. The eluted proteins were separated by SDS-PAGE for Western blotting. Anti-Myc (Sigma) and -MBP (NEB) antibodies were used in this assay.

### Chromatin immunoprecipitation (ChIP) analysis

Constructs carrying *p35S::ATBMI1C-YFP* were transformed into wild-type plants, and homozygous lines were identified at the T3 generation for ChIP analysis. ChIP was performed as described [40] using ten-day-old seedlings grown on MS medium under LD conditions. Anti-H3K27me3 and -H3K4me3 antibodies were purchased from Upstate Biotechnology. Quantitative real-time PCR was performed to detect the *FLC* regions harboring H3K27me3 and H3K4me3 modifications with the primer pairs shown in Table 5. The locations of the primer pairs are given in Figure 8C. All ChIP assays were performed three times using at least three biological replicates.

**Table 5:** Primers used in this study.

Primer name	Primer sequence (5′ to 3′)
*ATBMI1C*-SpeI-F	ACTAGTTGGGAATCGGAGAGAAAGATGTTA
*ATBMI1C*-BamHI-R	GGATCCCTTCAGAGGCAGAGCCAGAGTCAGAG
*ATBMI1C*-p1969-F	CTGCAGCGTACGACCATTCAATTCTTGC
*ATBMI1C*-p969-R	TCTAGACTTTCTCTCCGATTCCCAAAC
*SUC2*-F	AAAATCTGGTTTCATATTAATTTCA
*SUC2*-R	ATTTGACAAACCAAGAAAGTAAGA
*KNAT1*-F	GATCTAGAGCCCTAGGATTTGA
*KNAT1*-R	ACCCAGATGAGTAAAGATTTGAG
*AtBMI1C*-RTF	CATGCCTTGCTTGTCCAATC
*AtBMI1C*-RTR	GCTTCGTCCAATCCATTGTC
*Actin2/7*F	AGGCACCTCTTAACCCTAAAGC
*Actin2/7*R	GGACAACGGAATCTCTCAGC
*Actin2/7*rtF	GGTGTCATGGTTGGTATGGGTC
*Actin2/7*rtR	CCTCTGTGAGTAGAACTGGGTGC
*FLC*-F	CCTCTCCGTGACTAGAGCCAAG
*FLC*-R	AGGTGACATCTCCATCTCAGCTTC
*FT*-F	ACTATAGGCATCATCACCGTTCGTTACTCG
*FT*-R	ACAACTGGAACAACCTTTGGCAATG
*FLC*P1F	GCATTAGGTTGTTCCCTCCAAAC
*FLC*P1R	GCCCTACCCATGACTAACGTGAG
*FLC*P2F	GTTCGGGAGATTAACACAAATAATAAAGG
*FLC*P2R	GAAAACAAGCTGATACAAGCATTTCAC
*FLC*P3F	TGGGGGTAAACGAGAGTGATG
*FLC*P3R	GCAATAGTTCAATCCGTATCGTAGG
*FLC*B1F	TGTTCTCAATTCGCTTGATTTCTAGT
*FLC*B1R	GCCCGACGAAGAAAAAGTAGATAG
*FLC*B2F	CGAGCACGCATCAGATCG
*FLC*B2R	GGCGGATCTCTTGTTGTTTCTC
*FLC*B3F	GACGTGCATATACAAATCCAAGAGAAC
*FLC*B3R	CTTTGAATCACAATCGTCGTGTG
*FLC*B4F	CCTCTCCGTGACTAGAGCCAAG
*FLC*B4R	CTTCAACATGAGTTCGGTCTGC
*FLC*B5F	CCTTGGATAGAAGACAAAAAGAGAAAGTG
*FLC*B5R	AGGTGACATCTCCATCTCAGCTTC

### Western blotting

Total protein was extracted using protein extraction buffer (50 mM Tris-HCl [pH 7.5], 150 mM NaCl, 10 mM MgCl_2_, and 1% NP-40) containing complete protease inhibitor cocktail (Roche), separated by SDS-PAGE, and probed with anti-GFP and -tubulin antibodies, respectively.

Histone-enriched protein extracts were prepared as described previously using ten-day-old seedlings [41]. Nuclei were isolated using extraction buffers I (0.4 M sucrose, 10 mM Tris-HCl [pH 8.0], 10 mM MgCl_2_, 5 mM β-ME, 0.1 mM PMSF, and complete protease inhibitor cocktail), II (0.25 M sucrose, 10 mM Tris-HCl [pH 8.0], 1% Triton X-100, 10 mM MgCl_2_, 5 mM β-ME, 0.1 mM PMSF, and complete protease inhibitor cocktail), and III (1.7 M sucrose, 10 mM Tris-HCl [pH 8.0], 0.15% Triton X-100, 2 mM MgCl_2_, 5 mM β-ME, 0.1 mM PMSF, and complete protease inhibitor cocktail) in turn. The chromatin was treated overnight with 0.4 M H_2_SO_4_ at 4°C and the proteins were precipitated with 25% trichloroacetic acid. The precipitate was washed three times with acetone, air-dried, and resuspended in 4 M urea. The histone-enriched protein extracts were separated by 15% SDS-PAGE, transferred to a PVDF membrane, and probed with anti-ubiquitin and -H3 antibodies, respectively.

### Alignment and phylogenetic analysis

A phylogenetic tree of RING domain-containing proteins and RING domains from different organisms was constructed using MEGA4 software [42]. The RING domains of the RING domain-containing proteins were aligned using Jalview through ClustalW [43].
